# Fibromatosis involving pelvic floor muscles

**DOI:** 10.1259/bjrcr.20150239

**Published:** 2016-07-28

**Authors:** Aleksandra Stankiewicz, Nelesh N Jeyadevan

**Affiliations:** Department of Radiology, Croydon University Hospital, Croydon, UK

## Abstract

Fibromatosis or desmoid tumour is a benign fibroblastic proliferation with aggressive infiltrative growth. High incidence of recurrence is noted after incomplete resection of the involved margins of the lesion. Pelvic fibromatosis is a rare condition and usually affects females. Patients frequently complain of pelvic pain, which can mimic gynaecological abnormalities. A case of pelvic fibromatosis involving pelvic floor muscles with unchanged appearance during 5 years of follow-up is presented.

## Clinical presentation and differential diagnosis

In 1991, a 29-year-old female was referred by a general practitioner to the gynaecology clinic with a several-month history of diminishing periods, deep dyspareunia and increasing difficulty and pain with defaecation. The patient felt like had a hard lump in the vagina. Vaginal examination revealed a firm, fixed tumour arising from the ischiorectal fossa suggesting a bony tumour, but no X-ray abnormality was seen.

## Investigation/imaging findings

Review of the CT scan report showed the presence of a 12-cm oval mass in the posterior and right lateral aspect of the pelvis that extended into the sacrosciatic notch with some obliteration of the intergluteal fat but with no localized invasion of the glutea. The right obturator internus muscle was not seen separately from the mass. The heterogeneously enhancing mass extended into the ischiorectal fossa towards the pelvic floor and was displacing the perineal body to the left. The bladder, uterus and rectosigmoid were also displaced and compressed by the mass.

Unfortunately, images of the CT scans were not available, as the analogue films from that era had been destroyed and subsequent images were digital. No record of digitization of the old CT images could be traced.

A needle biopsy was performed and showed that the tumour consisted of spindle cells rich in collagen fibres without cytological atypia and it was confirmed as fibromatosis (desmoid tumour).

## Treatment/outcome/follow-up

The patient was prescribed chemotherapy—methotrexate 50 mg and vinblastine 10 mg i.v. weekly. Repeat CT scan in July 1992 showed no objective change in the longstanding enhancing pelvic mass. The patient continued with the treatment. On vaginal examination in 1996, the mass appeared significantly smaller in size, measuring 8 × 4 × 4 cm. It was present in the paravaginal area and no involvement of the rectal or vaginal wall was noted. It was found during surgery that the right obturator internus muscle had been infiltrated by the desmoid tumour. Therefore, only incomplete resection of the mass was performed.

After the surgery, there was a significant worsening of the patient's longstanding problem with defacation, leading to continuous use of laxatives. This was thought to be related to the surgery.

A report of CT scan from August 2000 showed that the mass was continuing to expand the obturator internus muscle and extending to the right piriformis muscle, down to the level of the perineum. Infiltration of the pouch of Douglas was also seen. The mass was felt to be too close to the anal sphincters, and therefore only conservative management was offered with annual follow-up appointments. The appearances were stable, and on physical examination, only a small nodule on the posterior fornix was noted.

**Figure 1.**
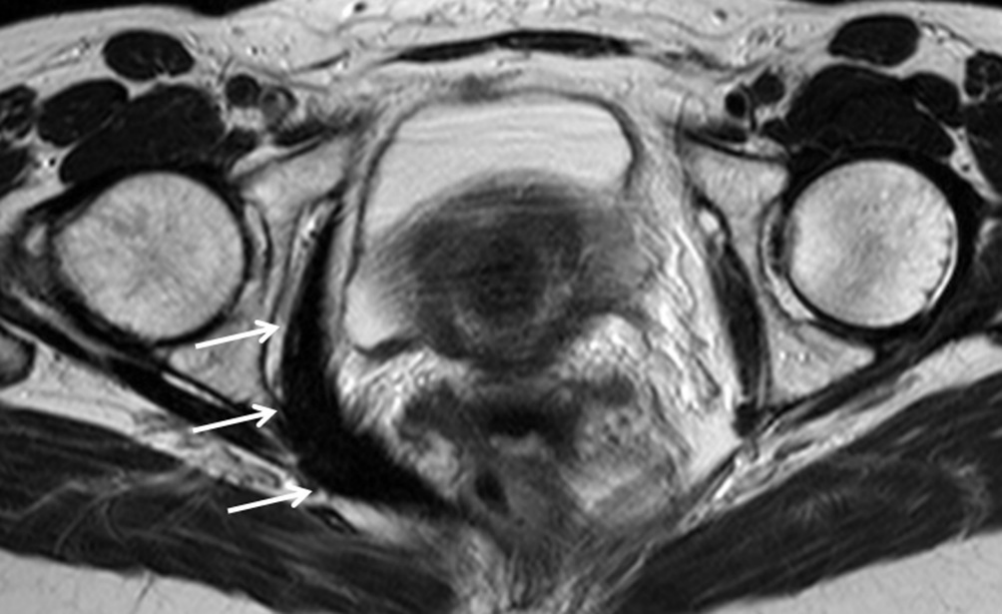
*T*_2_ weighted axial image showing abnormal low signal of the right obturator internus muscle (arrows), which is consistent with fibromatosis.

**Figure 2.**
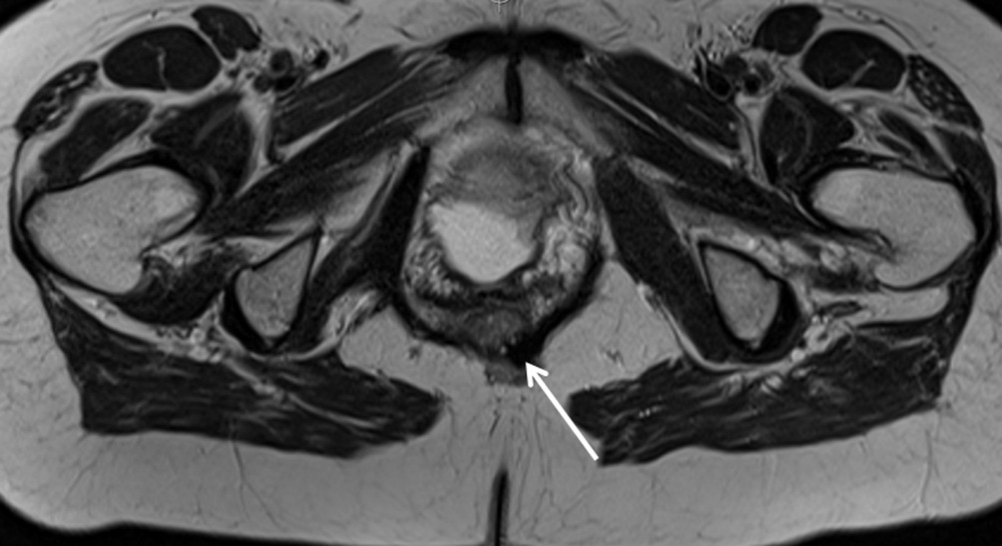
*T*_2_ weighted axial image showing involvement of the levator ani muscle and parasphincteric focus (arrow) of fibormatosis.

The patient underwent an MRI in 2009, which showed a focal low signal abnormality on *T*_1_ and *T*_2_ weighted, and short tau inversion-recovery sequences involving the right obturator internus muscle and which extended posteriorly inferior to the right piriformis muscle along the right sacral ligament to the S5–S6 level on the right side of the sacrum. The low signal abnormality was also seen along the obturator internus muscle posterolaterally and the right levator ani muscle (Figure 1). There was also a low signal focus seen involving the right aspect of the anal sphincters and left levator ani muscle (Figure 2). The appearances were consistent with pelvic floor muscle fibromatosis with parasphincteric focus. An enlarged uterus was also noted.

In 2011, the patient underwent laparoscopy, which revealed endometriosis in the uterosacral ligament. Subsequently, laparoscopic hysterectomy and bilateral salpingo-oophorectomy was performed. Since then, the patient has been complaining of increased pelvic and right hip pain, and difficulty with defecation, which were thought to be due to adhesions and known fibromatosis.

**Figure 3.**
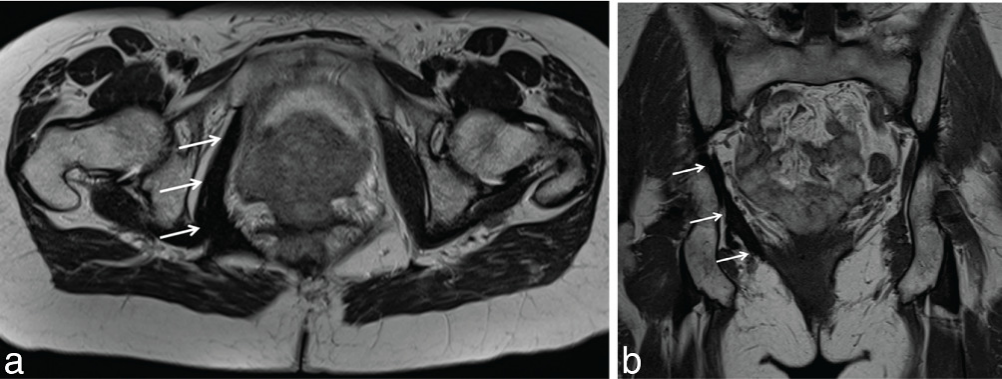
*T*_2_ weighted axial (a) and coronal (b) images. Unchanged appearances of pelvic fibromatosis involving pelvic floor muscles (arrows).

In 2014 she was referred for an MRI, which showed unchanged appearance of the pelvic floor fibromatosis involving the right obturator internus and piriformis muscle with parasphincteric focus (Figure 3a,b).

## Discussion

Aggressive fibromatosis or desmoid tumour is an infiltrating proliferation of fibroblasts without any evidence of inflammation. As it originates from the musculoaponeurotic structures,^1^ it can arise anywhere in the body, even in the most uncommon locations such as the vulva.^2^ However, the most common locations include paravertebral musculatures and anterior abdominal wall, particularly in relation to surgical scars, abdomen^3^ (involving mesentery or retroperitoneum) or pelvis.^1,4–6^

Intrapelvic fibromatosis is rare and involvement of the pelvic floor is uncommon, which is also reflected in the literature, as not many cases have been described.

Desmoid tumours occur more frequently in females of child-bearing age (female to male ratio of 3 : 1), occurring most commonly in the third or fourth decade of life.^1^ The aetiology of fibromatosis is not clear; however, trauma, abdominal surgery, drugs, irradiation, genitourinary inflammation or Gardner’s syndrome are thought to be causative factors.^7–11^ In this case, the patient did not have any of these conditions; therefore, this would represent a case of primary or sporadic fibromatosis. The primary form is extremely rare and is defined as fibroblastic proliferation with no relation to the patient’s medical or surgical history.^7^ The diagnosis of primary fibromatosis is difficult to establish preoperatively, especially in patients with no medical history.

Initially, the patient underwent chemotherapy and surgical resection of the tumour, which is considered the ideal treatment for this type of fibromatosis.^12^ However, wide surgical resection with tumour-free margins could be achieved in only 50% of cases.^1,7,11^ It is noted that even in cases with negative margins of resection, the incidence of local recurrence varies between 13% and 68%.^12,13^ In our case, the pelvic floor muscles were initially infiltrated by the tumour, which prevented complete resection. No further surgical resection was considered appropriate, as imaging appearances had remained stable during the follow-up years. Moreover, the parasphincteric focus of fibromatosis was found to be too close to the anal sphincters and any intervention could have led to sphincter injury, and consequently faecal incontinence.

MRI typically shows isointense features on *T*_1_, low signal on *T*_2_ and avid enhancement on *T*_1_ weighted images with fat suppression techniques. The signal appearances are not diagnostic of fibromatosis but are characteristic. In cases of suspicion of recurrence, MRI is the imaging modality of choice.^1^ However, recent advances in ultrasound imaging, the emergence of high frequency transducers and automatic three dimensional (3D)/four dimensional acquisition, allow precise assessment of the pelvic floor.^14–16^ These ultrasound techniques are commonly used in gynaecological, urogynaecological or proctological practices, but are not yet so widely applied by radiologists. Various studies have shown the usefulness of 3D high frequency endoanal ultrasound imaging in the evaluation of the normal anatomy and injuries of the anal sphincters.^14,15^ 3D endovaginal and transperineal ultrasound imaging are well-recognized techniques in the assessment of pelvic floor structures, levator ani muscle and levator hiatus,^14,15,17^ with good interobserver reliability.^16,18,19^ It would be worthwhile to assess their usefulness in the diagnosis of such a rare condition as pelvic fibromatosis.

In our case, MRI showed a tumour in relation to the right levator ani muscle and right obturator internus muscle and focus of fibromatosis in the perianal region, determining its further conservative management. These appearances have remained stable during 5 years of follow-up.

## Learning points

1. Primary pelvic floor fibromatosis is extremely rare; however, it should be considered in females of child-bearing age with pelvic symptoms.
2. Owing to lack of definitive therapy, desmoid tumours are exceedingly difficult to treat and require an interdisciplinary approach for best management.
3. MRI is the method of choice when fibromatosis and recurrence are suspected.
4. Novel ultrasound techniques with the use of high frequency transducers, 3D data acquisition and endoanal/endovaginal/transperineal approach enable precise assessment of the pelvic floor structures.

## Consent

Informed consent has been obtained.
